# The Role and Mechanisms of Selenium Supplementation on Fatty Liver-Associated Disorder

**DOI:** 10.3390/antiox11050922

**Published:** 2022-05-08

**Authors:** Lin Xu, Yuanjun Lu, Ning Wang, Yibin Feng

**Affiliations:** School of Chinese Medicine, Li Ka Shing Faculty of Medicine, The University of Hong Kong, Hong Kong 999077, China; xlcarol@hku.hk (L.X.); yjlu@connect.hku.hk (Y.L.); ckwang@hku.hk (N.W.)

**Keywords:** selenium, NAFLD, hepatic fibrosis, hepatocellular carcinoma, oxidant defense, inflammation

## Abstract

Non-alcoholic fatty liver disease (NAFLD) is the most frequent chronic liver disease without effective therapy. Selenium, as an essential trace element for humans, is notable for its antioxidant properties. The previous study shows that selenium levels in NAFLD patients are lower than normal ones. Selenium supplementation can effectively alleviate metabolic disorders by relieving anti-oxidative stress and anti-inflammatory regulation. However, the correlation between selenium and NAFLD has not been fully clarified. Herein, we review the current studies on selenium in regulating the different stages of NAFLD and summarize relevant clinical trials to highlight the potential roles of selenium in NAFLD treatment.

## 1. Introduction

Selenium is an essential trace element in human nutrition, named after the Greek word “Selene”, which was initially characterized as a by-product of sulphuric acid manufacturing in 1817 [1]. The earliest evidence of selenium’s biological activity was identified in microbes in 1954 [2]. The endogenous level of selenium in plants varies from place to place and among different populations due to the selenium content in the soil being changed in different geographical regions. Selenium is a trace element that is vital for human health. Poor selenium status leads to leukonychia, leukoderma, poor wound healing, and gestational disorders [3,4]. Selenium is also found in the body’s natural defense system against infection and oxidative stress [3]. An excessive amount of selenium, on the other hand, can be harmful to people. For adult men and women over 19, the Recommended Dietary Allowance (RDA) is 55 μg per day, and for all individuals, including pregnant or breastfeeding women, the Tolerable Upper Intake Level (UL) for selenium is 400 μg per day [5]. Dietary selenium is obtained chiefly from the following food groups: meat and fish, eggs and dairy products, and bread and cereals [6]. For clinical application, selenium can be used to treat erosive oral lichen planus [7] and relieve side effects in cancer patients after chemotherapy and radiotherapy [8]. In addition, selenium nanoparticles can be used as cancer-targeted drugs [9]. Selenium is commonly found in multivitamin/multimineral supplements and as a stand-alone supplement, most frequently in the form of selenomethionine or selenium-enriched yeast (produced in a high-selenium culture) or as sodium selenite or sodium selenate. Compared to selenite, the human body can absorb more from selenomethionine, reaching 90% [10]. However, the difference between various selenium types in their bioavailability has not been fully investigated. In one of the studies, ten groups of selenium-deficient subjects were randomly assigned to receive a placebo, 200 or 600 μg/day of selenium in the mentioned supplementary form, such as selenomethionine, sodium selenite, or high-selenium yeast. Based on urine excretion, selenomethionine had the highest bioavailability, while selenite had the lowest [11].

Non-alcoholic fatty liver disease (NAFLD) is the most frequent obesity-related metabolic syndrome in the liver, defined by the excessive accumulation of hepatic lipids in the absence of alcohol abuse [12]. Obesity and type 2 diabetes are both known to increase the risk of NAFLD. Other risks include being overweight, having metabolic syndrome with the following medical conditions: abdominal obesity, high blood pressure, high blood sugar, high serum triglycerides, and low serum HDL cholesterol, and having a high-fructose diet. With a 15–40% high incidence in the general population, it has become the most common chronic liver disease [13]. By 2030, the global prevalence is expected to climb by 21% [14]. Furthermore, fatty liver can progress to more advanced hepatic metabolic disorders such as non-alcoholic steatohepatitis, fibrosis, cirrhosis, and hepatocellular carcinoma [15]. Indication of lifestyle changes, including diet and physical activity, is the most effective strategy to treat NAFLD, either non-alcoholic fatty liver (NAFL) or non-alcoholic steatohepatitis (NASH). For pharmacological treatments, vitamin E or pioglitazone can only be used in nondiabetic patients with biopsy-proven NASH [16].

NAFLD is a multifactorial metabolic disorder for which there are currently no Food and Drug Administration (FDA)-approved therapies. Drugs for the therapy of NAFLD include antioxidants, insulin sensitizers, hypoglycemic, and lipid-lowering agents, all of which are used to alleviate the symptoms. Recent studies have discovered that selenium levels in patients with NAFLD are relatively lower than those in healthy individuals. Such selenium reduction is frequently observed in patients with advanced hepatic metabolic diseases such as hepatitis, cirrhosis, and liver cancer [17]. However, the mechanism of selenium has not been entirely explained. In this review, the significance of selenium in the pathological progression of NAFLD and the underlying regulatory mechanism are the focuses of this review, and we provide a novel insight into the implication of selenium in NAFLD treatment.

## 2. Regulatory Mechanisms of Selenium

**Figure 1:**
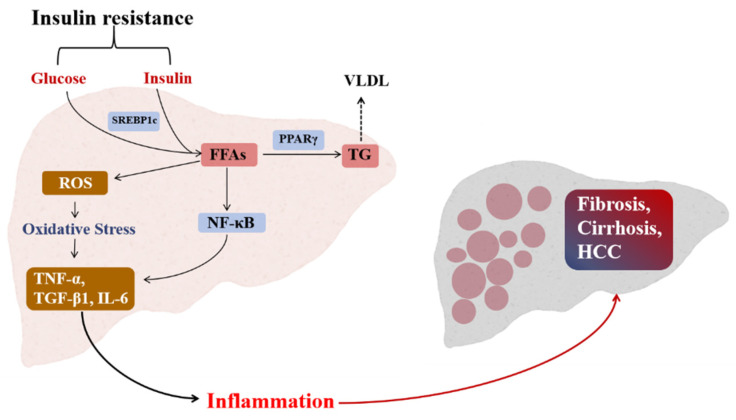
An overview of the pathophysiological mechanisms that oxidative stress and inflammation in the development of non-alcoholic fatty liver disease. Briefly, free fatty acid (FFA) flux could activate the transcriptional factors such as peroxisome proliferator-activated receptor gamma (PPARγ). In an aggravated state, this process promotes the increased production of reactive oxygen species (ROS) and aberrant levels of pro-inflammation markers such as tumor necrosis factor-alpha (TNF-α), the nuclear factor kappa B (NF-κB) and transforming growth factor beta (TGFβ-1). SREBP1c = sterol regulatory element-binding protein; TG = triglycerides; ROS = reactive oxygen species; IL-6 = interleukin 6; VLDL = very low-density lipoproteins.

The pathogenesis of NAFLD is extremely complex and has not yet been fully understood. What is generally recognized by the medical community is the hypothesis of “two-hit”, jointly proposed by Day and James in 1998 [18], which has become the main theory to clarify the mechanism of the disease. According to this hypothesis, lipid accumulation in the liver was caused by insulin resistance (IR), forming the “first hit”. On this basis, the damage to liver cells was further accelerated and aggravated through inflammatory cytokines, mitochondrial dysfunction, and oxidative stress. It makes up the “second hits” and eventually leads to steatohepatitis or fibrosis. These factors affecting NAFLD etiology are neither mutually exclusive nor independent. Rather than that, it coordinates and cooperatively promotes the advancement of NAFLD. For instance, obesity is related to elevated levels of inflammatory markers and oxidative stress, as well as an exacerbated inflammatory response. Cytokines can either cause direct harm to the liver or impair its function indirectly through their effects on oxidative stress and inflammatory responses. Relative pathophysiological mechanisms that cause oxidative stress and inflammation in NAFLD are shown in Figure 1.

### 2.1. The Effect of Selenium on Insulin Resistance

Insulin plays a critical role in glucose homeostasis by balancing the synthesis of glucose by the liver and glucose absorption by muscle and adipose tissue. Insulin resistance impairs the antilipolytic action of adipose tissue, resulting in increased fatty acid release [19]. Insulin resistance is associated with elevated insulin levels, which stimulate hepatic triglyceride production in the setting of increased lipolysis and/or fat intake [20]. The changes in selenium status seem to play a key role in the development of insulin resistance [21]. After 4 weeks of treatment, sodium selenite was found could increase both mRNA and protein levels of the glucagon-like peptide-1 receptor (GLP-1R), insulin receptor substrate-1 (IRS-1), and preproinsulin mRNA levels in the liver of diabetic rats [22]. GLP-1 tends to increase glycogen synthesis, hence decreasing hepatic glucose production [23], and it has also been found to affect insulin synthesis and secretion, as well as proliferation, differentiation, and apoptosis of pancreatic β cells [24]. What is more, the number of endocrine islets increased after selenium treatment, and the cellularity and viability of pancreatic tissue were restored [25], further suggesting that selenium may have a role in the physiological effects of incretin hormones. In addition, a study found that in diabetic rats given sodium selenite intraperitoneally for 14 days, glucose absorption by peripheral organs and adipocytes increased [26]. This was most likely due to selenium’s impact on the insulin receptor, which enhanced insulin sensitivity and alleviated hyperglycemia by allowing for the regulation of hepatic glucose production.

### 2.2. The Regulation of Selenoproteins as Endogenous Antioxidant Defense

Living organisms produce reactive oxygen species (ROS) on account of regular cellular metabolism and extrinsic effects. ROS are extremely reactive chemicals that are able to damage and change the functioning of cell components such as carbohydrates, nucleic acids, lipids, and proteins. Oxidative stress (OS) refers to a shift in the equilibrium of oxidants and antioxidants in favor of oxidants. The redox state regulation is crucial for cell survival, activation, proliferation, and organ function [27]. NAFLD is strongly associated with the presence of oxidative stress. In NAFLD patients, elevated OS has been widely reported [28]. When oxidative stress is elevated, as occurs in NAFLD, lipid peroxidation would subsequently arise and lead to lipid hydroperoxide production. Those lipid hydroperoxides, together with increased cytokines produced by Kupffer cells and hepatocytes, can cause direct damage to liver cell membranes, enhance intrahepatic inflammation, and induce fibrosis in the liver. Changes in intracellular redox status and oxidative changes in proteins are the two main mechanisms of action in current ROS signaling theories. Thiol redox systems, primarily glutathione and thioredoxin, mitigate intracellular oxidative stress by decreasing H_2_O_2_ and lipid hydroperoxides in the first mechanism [29].

Selenium, in the form of selenoproteins, serves crucial biological activities in organisms. From the human genome, 25 selenoproteins have been discovered thus far [30], including glutathione peroxidase (GPxs), and thioredoxin reductase (TrxRs), as well as several uncharacterized selenoproteins. The relevance of selenium is highlighted by its involvement as a component of several important antioxidants, as well as its unique redox properties and utilization in antioxidant enzymes such as GPXs and TrxRs [31]. GPxs belong to the family of antioxidant enzymes. Cytosolic GPx, gastrointestinal-specific GPx, plasma GPx, and phospholipid hydroperoxide GPx, representing GPx1 to 4, respectively, are the four main GPxs. Their primary function is to neutralize intracellular and extracellular hydrogen peroxide and organic peroxides, affecting signaling and preventing oxidative damage. Because the enzymatic activity is proportional to selenium intake, selenium deficiency is strongly associated with the oxidation of the body. GPx1–3 are responsible for the reduction of hydrogen peroxide and organic hydroperoxides, whereas GPx4 is responsible for the reduction of phospholipid hydroperoxides and cholesterol hydroperoxides directly [6]. The increased vulnerability of animals that lacked GPx1 and GPx2 to oxidative stress indicates that GPx1 and 2 have well-defined antioxidant activities [32]. The transgenic mice with GPx1 deficiency or overexpression showed additional regulatory functions of GPx1 in reactive oxygen and nitrogen species and insulin secretion and insulin resistance [33]. TrxRs are pyridine nucleotide sulfide oxidoreductases belonging to the flavoprotein family. TrxRs regulate cellular proliferation, survival, and apoptosis by regulating thioredoxin activity and redox status. They also play an important part in oxidative stress’s biological response via the efficient removal of various ROS [34]. The thioredoxin (Trx) system functions in numerous physiological signaling cascades by regulating the activity of transcription factors with crucial cysteines in their DNA-binding domains, such as nuclear factor kappa B (NF-κB), activator protein-1 (AP-1), p53, and the glucocorticoid receptor [35]. The redox regulation by thioredoxin reductase (TXNRD) systems could affect the biological response to oxidative stress, cell proliferation, as well as inflammatory modulation [36].

### 2.3. The Modulation of Selenium on Inflammation

Selenium and selenoproteins are crucial for establishing or enhancing immunity. They can also play a role in immunological modulation, which is important for avoiding overreactions that can lead to autoimmunity or chronic inflammation. In addition, inflammation is also widely recognized as a key factor in the development and progression of NAFLD and liver damage. Studies showed that supplementing selenium to selenium-deficient macrophages could reduce the expression of two pro-inflammatory genes, cyclooxygenase-2 (COX-2) and tumor necrosis factor-alpha (TNF-α), which were induced by lipopolysaccharide (LPS) [37]. Vunta et al. found that selenium supplementation increases the production of 15-deoxy-Delta12,14-prostaglandin J2 (15d-PGJ2), which is the endogenous inhibitor of IkappaB-kinase beta (IKKbeta) activity, as an adaptive response to protect cells from pro-inflammatory gene expression triggered by oxidative stress [38]. Therefore, selenium contributes to the anti-inflammatory function of immune cells by modulating the expression of pro-inflammatory genes. In vivo, selenium can modulate glutathione peroxidase activity. Selenium can also suppress NF-κB activation and up-regulate the half-life of I kappa B alpha [39]. Selenoprotein S (SEPS1) is also an important selenoprotein, localizing to the endoplasmic reticulum and acting as an anti-inflammatory factor. In the pancreas and blood vessels, it provides antioxidant protection and has anti-ER stress actions, but in the liver, adipose tissue, and skeletal muscle, it increases the onset and development of insulin resistance [40]. SEPS1 displays regulatory functions in stress response and inflammation control. In macrophage cells, the suppression of SEPS1 by short interfering RNA increased the release of IL-6 and TNF-α, which reveals a direct mechanistic relationship between SEPS1 and the production of inflammatory cytokines and implies that SEPS1 plays a role in mediating inflammation [39].

## 3. The Role of Selenium on the Progression of NAFLD

### 3.1. Selenium and Lipid Metabolism in the Liver

For circulating lipids, the liver serves as a metabolic hub. The net retention of lipids within hepatocytes, usually in the form of triglycerides, is the first step in the initiation of fatty liver [41]. The major pathways of lipid metabolic abnormalities are related to the changes in lipid uptake, lipogenesis, lipolysis, lipophagy, and expression of hepatic lipoproteins. Speckmann B et al. showed that selenium supplementation increased the hepatic mRNA expression of PPARα in the mice [42], which can mediate the prevention of triglyceride accumulation [43]. For patients with gestational diabetes mellitus, taking selenium yeast 200 μg per day as selenium supplements for 6 weeks upregulated peroxisome proliferator-activated receptor gamma (PPARγ) and glucose transporter 1 (GLUT-1), which inhibited lipogenesis [44]. Moreover, selenium deficiency could decrease the expression of intracellular antioxidant enzymes, including Superoxide dismutase-1 (SOD1) [45], which results in the development of fatty liver [46]. Furthermore, supplementing with selenium and magnesium inhibits the mRNA expression level of hepatic lipogenesis genes liver X receptor alpha (LXRα), SREBP-1c, and FASN (fatty acid synthase) to reduce the hepatic total cholesterol (TC) and attenuate liver steatosis in the rats fed by high-fat diet [47]. Moreover, a study found zinc and selenium co-supplementation could significantly reduce the level of serum triglyceride and TC. The fat accumulation was considerably reduced in high-fat diet-fed rats [48].

### 3.2. Selenium and Inflammatory Response in Liver

The management of inflammatory processes in NAFLD may prevent the development of NASH to liver fibrosis [49]. It has been proven that selenium deficiency could initiate inflammation by activating the NF-κB pathway through multiple mechanisms at an organ level [50]. Selenium supplementation can reduce pro-inflammatory cytokines and the expression of inflammation-related proteins in the liver, such as TLR4, NF-κB, JNK, and p38, as well as upregulating heme oxygenase-1 (HO-1) to reduce the inflammatory response. Al-Dossari et al. found that the selenium treatment (0.1 mg/kg/d) mitigated lipopolysaccharide (LPS)/Diclofenac (DCL)-induced injury in the liver through suppressing the LPS-induced TLR4 signaling pathway and boosted antioxidant defenses to reduce oxidative stress in rats [51]. In the mercury chloride (HgCl_2_)-induced hepatotoxicity animals, selenium alleviated the inflammation by blocking the NF-κB/NLR family pyrin domain containing 3 (NLRP3) inflammasome signaling pathway. Moreover, selenium prevented HgCl_2_-induced liver lipid accumulation and dyslipidemia by regulating gene expression related to lipid and glucose metabolism [52].

### 3.3. Selenium and Hepatic Fibrosis Formation

Liver fibrosis is common in the majority of types of chronic liver diseases, and its obvious characteristic is a condition in which extracellular matrix proteins, such as collagen, build up excessively and generate fibrous scars. According to a study, serum selenium levels are inversely related to the risk of advanced liver fibrosis, especially in older individuals, Caucasians, and females. Increased selenium levels reduced all-cause mortality [53]. In the N-nitrosodimethylamine (NDMA)-driven hepatic fibrosis rat model, reduced selenium and glutathione peroxidase can be observed to contribute to the weakening of cellular antioxidant defense to aggravate the progression of hepatic fibrosis [54]. In carbon tetrachloride (CCl_4_-induced liver fibrosis, selenium supplementation in the form of sodium selenite in the drinking water boosted selenium-dependent glutathione peroxidase activity and reduced malondialdehyde in liver tissues. Selenium can lower the number of collagen-producing stellate cells and increase collagen degradation to inhibit fibrosis [55]. Zhang et al. observed that compared to conventional green tea, selenium-enriched green tea products had a more pronounced improvement in liver extracellular matrix deposition, scar formation, and peripheral 5-hydroxytryptamine signals [56]. Selenium-enriched probiotics contain 10 g of selenium per milliliter, of which more than 90% is organic Se significantly reduced the fibrosis-related gene expression of α-smooth muscle actin, collagen, transforming growth factor-beta (TGF-β1), and TIMP-1, which involved the apoptosis of stimulated hepatic stellate cells [57]. Other studies show that selenium-glutathione-enriched probiotics, which contained 38.4 μg/g of organic selenium and 34.1 mg/g of intracellular glutathione, attenuated liver fibrosis by increasing the hepatic silent information regulator 1 (SIRT1) and MAPK pathway and involved the down-regulation of oxidative stress, endoplasmic reticulum stress, and inflammation in CCl_4_-treated rats [58]. Vitamin E and selenium in combination could have a synergistic effect on inhibiting CCl_4_-induced hepatic injury. They have been shown to limit hepatic stellate cell growth and increase apoptosis in activated hepatic stellate cells (HSCs) during the acute injury phase. Vitamin E and selenium can also significantly reduce the severity of hepatic fibrosis and facilitate the healing process [59].

### 3.4. Selenium and Liver Cirrhosis Development

Studies have long found that the plasma levels of selenium in patients with cirrhosis were lower than physical plasma concentrations and that they decrease in proportion to the severity of the cirrhotic condition [60]. In rats with carbon tetrachloride (CCl_4_)/ethanol-induced cirrhosis, the co-treatment of selenium and vitamin E significantly decreases the amount of hepatic fibrosis [61]. The study of thioacetamide-induced liver cirrhosis showed that sodium selenite supplementation (1 mg/kg b.w, i.p. for 12 weeks) reduced total bilirubin and ALT activity, as well as restored the antioxidant enzymes (SOD and GSH), MDA, and catalase activity [62]. However, selenium supplementation could not restore hepatic GPx and selenium to normal levels in selenium-deficient thioacetamide-induced cirrhosis mice [63].

### 3.5. Selenium and Hepatocellular Carcinoma

Hepatocellular carcinoma (HCC) is the most frequent type of primary liver cancer in adults, and it is now the leading cause of mortality in cirrhotic patients [64]. A meta-analysis shows the increased concentration of serum selenium could be related to a lower risk of HCC [65,66]. In CCl_4_-induced liver cancer, selenium was observed to decrease the elevation of alpha-fetoprotein (AFP) and TNF-α compared with control mice. The serum AFP is considered a tumor marker for liver cancer. Studies found that at the early stage of HCC, the low selenium levels caused the accumulation of lipid peroxides, which sped up the development of the disease by enhancing AP-1 activation and resulting in increased production of VEGF and IL-8. Therefore, selenium supplantation in the form of selenite could be investigated as chemoprevention or supplementary therapy for early HCC patients with low selenium levels [67]. Furthermore, pleomorphic adenoma gene like-2 (PLAGL2) overexpression has been linked to tumorigenesis. Yang et al. found that selenium sulfide (SeS2) suppressed C-MET/STAT3, AKT/mTOR, and MAPK signaling and prompted Bcl-2/Cyto C/Caspase-mediated intrinsic mitochondrial apoptosis in HCC cells in a PLAGL2-dependent manner by triggering Bcl-2/Cyto C/Caspase-mediated intrinsic mitochondrial apoptosis in a PLAGL2-dependent manner [68]. Thus, selenium shows a protective role against liver cancer [69]. The role of selenium in animal models has been summarized in Table 1.

## 4. Discussion and Conclusions

In this paper, the role of selenium in the development of NAFLD has been summarized. According to most of the evidence from experimental trials, selenium appears to have a beneficial effect on the improvement of hepatic steatosis, NASH, and fibrosis. At first, selenium can promote the upregulation of lipid oxidation enzymes as well as the downregulation of de novo lipogenesis enzymes, which may be used to combat steatosis [70]. Moreover, the antioxidant impact of selenium on the liver is at least in part through enhancing the activity of GPx, whose decrease leads to hepatic inflammation and liver fibrosis [71,72]. Selenium potentially suppresses the metalloproteinases, TNF-α, IL-6, TGF-β1, and other cytokines and growth factors involved in NAFLD etiology, which might help to reduce hepatic inflammation and fibrosis [71,73,74,75]. It has been postulated that Se supplementation in chronic inflammation recovers depleted hepatic Se levels and, by boosting selenoprotein biosynthesis, restores circulating Se levels, suppressing C-reactive protein production and finally attenuating the inflammatory response. However, most of the models are induced by CCl_4_ for manifesting hepatic inflammation and fibrosis; technically, it represents the toxic influence on the liver instead of the non-alcoholic fatty liver. Thus, the majority of animal research has been conducted on models that do not fully resemble human NAFLD. A randomized clinical trial revealed the relationship between selenium supplementation and insulin resistance. However, compared to the placebo group, the selenium group (200 µg/day) did not have a significant adverse effect on β-cell function or insulin sensitivity [76]. Since the in vitro and in vivo effects of selenium on NAFLD progression have been well outlined, the clinical trials of selenium warrant further estimation. In Korea, selenium levels were significantly lower in hepatitis and hepatoma patients compared to healthy controls, indicating a similar correlation between liver disease progression and decreases in Se levels, except for liver cirrhosis. Furthermore, the findings of significantly reduced Se levels in Korean hepatoma patients were validated in this investigation [77]. However, in China, higher plasma selenium levels were linked to a higher frequency of NAFLD and higher levels of fasting plasma glucose, post-loading plasma glucose, triglycerides, ALT, and AST. By triggering inflammation and infiltrating the liver, selenium exposure exacerbated liver damage [78].

Although selenium benefits the organism to some extent, an overdose of selenium can lead to severe effects. It is vital to be aware of its toxicity, most often attributable to excessive daily food and water intake. Selenium may take directly toxic effects by the modulation of ROS-related oxidative stress [79,80]. An outbreak of acute selenium poisoning in America was investigated; a high concentration of selenium in a liquid nutritional supplement containing 200 times (200 μg of selenium per fluid ounce) was found as the outbreak’s source. Diarrhea, weariness, hair loss, joint discomfort, nail discoloration or brittleness, and nausea are the most reported symptoms of its side effects. Fingernail discoloration and loss, weariness, and hair loss were among the signs that lasted 90 days or longer [81]. After ingesting 10 g of sodium selenite, a 75-years-old man was reported to suffer a cardiac arrest and die 6 h after ingestion [82]. Because of the limited range between therapeutic and dangerous amounts of selenium and because its effect depends on the form, dose, and type of therapy used, selecting the most effective supplement is a difficult task and needs further study. Additionally, though the supplement of selenium exists in different forms such as selenomethionine, selenium-enriched yeast, or sodium selenite or sodium selenate, there has been no investigation of how different kinds of selenium supplementation influence molecular signaling in vivo or how they affect treatment outcomes. Due to the restricted treatment window, the changes in effect and intake of selenium caused by the difference in form require further exploration in order to provide a more rigorous experimental basis for future therapeutic uses.

## 5. Challenges and Outlooks

In recent years, selenium has been promoted as a beneficial supplement for humans. A prudent selenium intake should be safe and useful for both healthy people and those suffering from metabolic diseases. It is expected that more clinically relevant research will be conducted in the future as attempts to understand the role of selenium in metabolic diseases continues to be made. Since the non-alcoholic fatty liver is a chronic disease, long-term observation is required in future clinical monitoring. In addition, the in vitro models of liver cirrhosis, liver fibrosis, and hepatocellular carcinoma in the late stage of NAFLD progression need to be further standardized so that the regulation of selenium on the overall progression of NAFLD can be precisely and fully evaluated rather than specific phenomena.

In conclusion, we conducted a thorough study of the involvement of selenium in a variety of non-alcoholic fatty acid metabolism pathways. It takes effect on the development of NAFLD by acting as an antioxidant and controlling the inflammatory response. Our study demonstrates the therapeutic potential of selenium supplementation in the treatment of metabolic disorders.

## Figures and Tables

**Table 1:** The role of selenium in animal models.

Component	Animal	Model	Route of Administration	Main Outcome	Ref
Sodium selenite	Rats	High-fat diet induced hyperlipidemic	Oral administration	Attenuated liver steatosis	[47]
Sodium selenite	Mice	Carbon tetrachloride (CCl_4_) induced hepatic fibrosis	Intraperitoneal injection	Decreased hepatic fibrosis after CCl_4_	[55]
Selenium-enriched green tea	Mice	Carbon tetrachloride (CCl_4_) induced hepatic fibrosis	Oral administration	Improved liver fibrosis	[56]
Selenium-glutathione-enriched probiotics	Rats	Carbon tetrachloride (CCl_4_) induced hepatic fibrosis	Oral administration	Attenuated liver fibrosis	[58]
Selenium and vitamin E	Rats	Carbon tetrachloride (CCl_4_) induced hepatic fibrosis	Oral administration	Decreased the degree of hepatic fibrosis and promote the recovery process	[59]
Selenium and vitamin E	Rats	Carbon tetrachloride (CCl_4_)/ethanol-induced cirrhosis	Oral administration	Decreased the amount of hepatic fibrosis	[61]
Sodium selenite	Rats	Thioacetamide induced cirrhosis	Intraperitoneal injection	Attenuated liver cirrhosis.	[62]
Sodium selenite	Mice	Thioacetamide induced cirrhosis	Oral administration Intraperitoneal injection	Could not restore hepatic glutathione peroxidase	[63]
Sodium selenite	Rats	Diethylnitrosamin (DEN) induced hepatocellular carcinoma	Oral administration	Accelerated the growth of hepatocellular carcinoma	[67]
Selenium	Mice	Carbon tetrachloride (CCl_4_) induced hepatocellular carcinoma	Oral administration	Protected against liver damage	[69]
